# 1-[3-(Anthracen-9-yl)-5-(pyridin-2-yl)-4,5-dihydro-1*H*-pyrazol-1-yl]ethanone

**DOI:** 10.1107/S160053681201505X

**Published:** 2012-04-13

**Authors:** Shi-Lu Zhang, Kun Huang, Da-Bin Qin

**Affiliations:** a Chemical Synthesis and Pollution Control Key Laboratory of Sichuan Province, School of Chemistry and Chemical Engineering, China West Normal University, Nanchong 637002, People’s Republic of China; b Department of Chemistry and Chemical Engineering, Sichuan University of Arts and Science, Dazhou 635000, People’s Republic of China

## Abstract

In the title compound, C_24_H_19_N_3_O, the pyrazoline ring adopts an envelope conformation with the C atom linking to the pyridine ring as the flap. The mean plane of the pyrazoline ring makes dihedral angles of 85.54 (4) and 81.66 (3)° with the pyridine ring and the anthracene ring system, respectively. In the crystal, mol­ecules are linked by C—H⋯O hydrogen bonds. In addition, weak π–π inter­actions [centroid–centroid distances = 3.695 (3)–3.850 (7) Å] are observed.

## Related literature

For applications of pyrazoline derivitives, see: Amir *et al.* (2008 ▶); Stell (2005 ▶). For the synthesis of the title compound, see: Lévai & Jekó (2006 ▶). For a related structure, see: Liu *et al.* (2008 ▶).
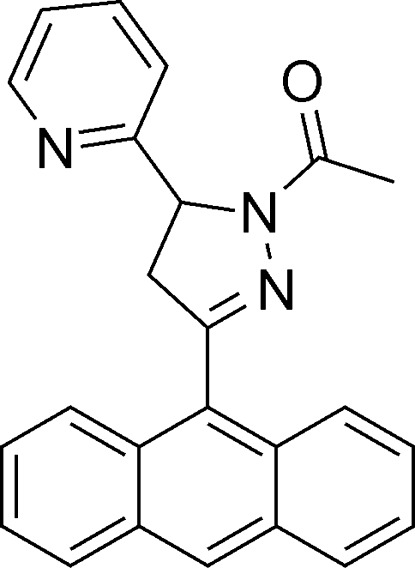


## Experimental

### 

#### Crystal data

C_24_H_19_N_3_O
*M* *_r_* = 365.42
Monoclinic, 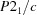

*a* = 10.1768 (8) Å
*b* = 23.6035 (18) Å
*c* = 7.9994 (7) Å
β = 109.134 (3)°
*V* = 1815.4 (3) Å^3^
*Z* = 4
Mo *K*α radiation
μ = 0.08 mm^−1^
*T* = 293 K
0.28 × 0.26 × 0.24 mm

#### Data collection

Rigaku Saturn diffractometer
Absorption correction: multi-scan (*CrystalClear*; Rigaku/MSC, 2004 ▶) *T*_min_ = 0.977, *T*_max_ = 0.980
10313 measured reflections
3544 independent reflections
3081 reflections with *I* > 2σ(*I*)
*R*_int_ = 0.033

#### Refinement

*R*[*F* ^2^ > 2σ(*F* ^2^)] = 0.040
*wR*(*F* ^2^) = 0.102
*S* = 1.02
3544 reflections
254 parameters
H-atom parameters constrained
Δρ_max_ = 0.22 e Å^−3^
Δρ_min_ = −0.24 e Å^−3^

### 

Data collection: *CrystalClear* (Rigaku/MSC, 2004 ▶); cell refinement: *CrystalClear*; data reduction: *CrystalClear*; program(s) used to solve structure: *SHELXS97* (Sheldrick, 2008 ▶); program(s) used to refine structure: *SHELXL97* (Sheldrick, 2008 ▶); molecular graphics: *SHELXTL* (Sheldrick, 2008 ▶); software used to prepare material for publication: *SHELXL97*.

## Supplementary Material

Crystal structure: contains datablock(s) global, I. DOI: 10.1107/S160053681201505X/hb6687sup1.cif

Structure factors: contains datablock(s) I. DOI: 10.1107/S160053681201505X/hb6687Isup2.hkl

Supplementary material file. DOI: 10.1107/S160053681201505X/hb6687Isup3.cml

Additional supplementary materials:  crystallographic information; 3D view; checkCIF report

## Figures and Tables

**Table 1:** Hydrogen-bond geometry (Å, °)

*D*—H⋯*A*	*D*—H	H⋯*A*	*D*⋯*A*	*D*—H⋯*A*
C12—H12⋯O1^i^	0.93	2.42	3.2745 (16)	153
C24—H24*A*⋯O1^ii^	0.96	2.58	3.5265 (16)	167

Symmetry codes: (i) 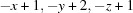; (ii) 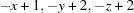.

**Fig. 1.**
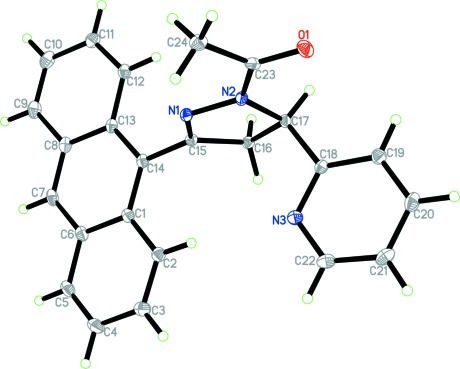
A view of the molecular structure of the title compound with displacement ellipsoids drawn at the 30% probability level.

**Fig. 2.**
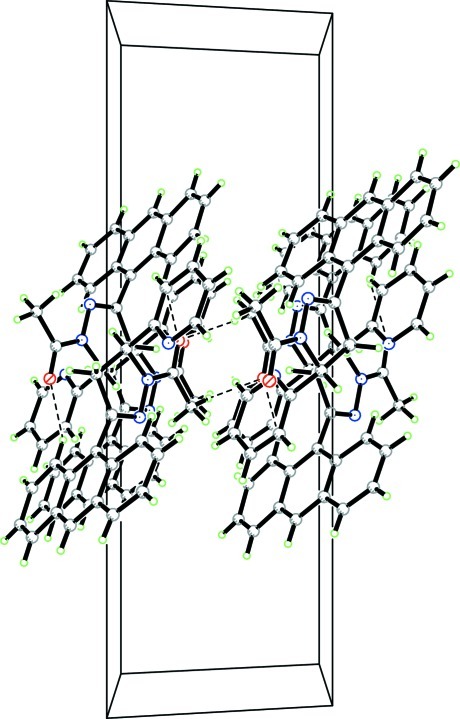
Part of crystal packing of the title compound, showing the molecules linked via C—H···O interactions (dashed lines). H atoms not involved in these interactions have been omitted for clarity. [symmetry codes: (i) 1 - x,2.5 + y,1.5 - z; (ii) 1 - x,2.5 + y,2.5 - z].

## supplementary crystallographic information

### Comment

Nowdays pyrazoline and its derivatives attract much attention of scientists due to its application in medication and coordination chemistry. (Amir *et al.*, 2008; Stell, 2005). Herein we report on the crystal structure of the title compound.

The molecular structure of the title compound is shown in Fig. 1. The mean plane of the Pyrzoline ring makes dihedral angles with the mean planes of the pyridine and anthracene rings of 85.54 (4)° and 81.66 (3)°, respectively.

In the crystal there are weak π–π interactions involving the pyridine, Pyrazoline and anthracene rings with centroid-centroid distances, *Cg*1···*Cg*2^i^, *Cg*2···*Cg*3^ii^ and *Cg*2···*Cg*4^ii^ of 3.695 (3), 3.768 (0) and 3.850 (7) Å, respectively [symmetry codes: (i) *x*, *y*, *z*; (ii) 1-*X*,-Y,-*Z. Cg*1 centroid of the Pyrazoline ring (N1, N2, C15—C17); *Cg*2 centroid of the pyridine ring(N3,C18—C22,); *Cg*3 centroid of ring (C1—C6); *Cg*3 centroid of ring (C1/C6/c7/c8/*c*13/*c*14)]. In addition, weak C—H···O hydrogen bonds interactions are observed (Table 1 and Fig. 2).

### Experimental

The title compound was prepared according to the reported procedures (Lévai *et al.*, 2006). Colourless prisms were obtained by recrystallization from ethyl acetate and petroleum ether (v:v = 1:1) solutoion.

### Refinement

H atoms were placed in calculated orientations and treated as riding atoms: C—H = 0.95 and 1.00 Å, with *U*_iso_(H) = 1.2*U*_eq_(C).

### Crystal data

**Table:**

C_24_H_19_N_3_O	*F*(000) = 768
*M**_r_* = 365.42	*D*_x_ = 1.337 Mg m^−^^3^
Monoclinic, *P*2_1_/*c*	Mo *K*α radiation, λ = 0.71070 Å
*a* = 10.1768 (8) Å	Cell parameters from 5004 reflections
*b* = 23.6035 (18) Å	θ = 2.3–27.9°
*c* = 7.9994 (7) Å	µ = 0.08 mm^−^^1^
β = 109.134 (3)°	*T* = 293 K
*V* = 1815.4 (3) Å^3^	Prism, colorless
*Z* = 4	0.28 × 0.26 × 0.24 mm

### Data collection

**Table:**

Rigaku Saturn diffractometer	3544 independent reflections
Radiation source: Rotating anode	3081 reflections with *I* > 2σ(*I*)
Graphite monochromator	*R*_int_ = 0.033
Detector resolution: 7.31 pixels mm^-1^	θ_max_ = 26.0°, θ_min_ = 2.3°
ω scans	*h* = −12→12
Absorption correction: multi-scan (*CrystalClear*; Rigaku/MSC, 2004)	*k* = −24→28
*T*_min_ = 0.977, *T*_max_ = 0.980	*l* = −7→9
10313 measured reflections	

### Refinement

**Table:**

Refinement on *F*^2^	Primary atom site location: structure-invariant direct methods
Least-squares matrix: full	Secondary atom site location: difference Fourier map
*R*[*F*^2^ > 2σ(*F*^2^)] = 0.040	Hydrogen site location: inferred from neighbouring sites
*wR*(*F*^2^) = 0.102	H-atom parameters constrained
*S* = 1.02	*w* = 1/[σ^2^(*F*_o_^2^) + (0.0546*P*)^2^ + 0.4522*P*] where *P* = (*F*_o_^2^ + 2*F*_c_^2^)/3
3544 reflections	(Δ/σ)_max_ = 0.001
254 parameters	Δρ_max_ = 0.22 e Å^−^^3^
0 restraints	Δρ_min_ = −0.24 e Å^−^^3^

### Special details

**Table:**

Geometry. All e.s.d.'s (except the e.s.d. in the dihedral angle between two l.s. planes) are estimated using the full covariance matrix. The cell e.s.d.'s are taken into account individually in the estimation of e.s.d.'s in distances, angles and torsion angles; correlations between e.s.d.'s in cell parameters are only used when they are defined by crystal symmetry. An approximate (isotropic) treatment of cell e.s.d.'s is used for estimating e.s.d.'s involving l.s. planes.
Refinement. Refinement of *F*^2^ against ALL reflections. The weighted *R*-factor *wR* and goodness of fit *S* are based on *F*^2^, conventional *R*-factors *R* are based on *F*, with *F* set to zero for negative *F*^2^. The threshold expression of *F*^2^ > σ(*F*^2^) is used only for calculating *R*-factors(gt) *etc*. and is not relevant to the choice of reflections for refinement. *R*-factors based on *F*^2^ are statistically about twice as large as those based on *F*, and *R*- factors based on ALL data will be even larger.

### Fractional atomic coordinates and isotropic or equivalent isotropic displacement parameters (Å^2^)

**Table:**

	*x*	*y*	*z*	*U*_iso_*/*U*_eq_	
O1	0.56753 (10)	1.02852 (4)	0.79960 (12)	0.0256 (2)	
N1	0.71048 (10)	0.91596 (4)	0.62713 (13)	0.0169 (2)	
N2	0.67267 (10)	0.97037 (4)	0.66237 (13)	0.0168 (2)	
N3	0.96041 (11)	1.02330 (5)	0.76512 (14)	0.0215 (2)	
C1	0.96119 (13)	0.85347 (5)	0.51788 (16)	0.0181 (3)	
C2	1.05711 (13)	0.88660 (5)	0.65379 (17)	0.0209 (3)	
H2	1.0268	0.9196	0.6931	0.025*	
C3	1.19243 (14)	0.87026 (6)	0.72619 (18)	0.0254 (3)	
H3	1.2528	0.8918	0.8163	0.030*	
C4	1.24244 (14)	0.82064 (6)	0.66544 (19)	0.0276 (3)	
H4	1.3355	0.8104	0.7139	0.033*	
C5	1.15428 (14)	0.78817 (6)	0.53671 (18)	0.0256 (3)	
H5	1.1883	0.7561	0.4971	0.031*	
C6	1.01021 (13)	0.80235 (5)	0.46098 (16)	0.0211 (3)	
C7	0.91593 (14)	0.76766 (5)	0.33701 (17)	0.0233 (3)	
H7	0.9479	0.7345	0.3007	0.028*	
C8	0.77543 (14)	0.78134 (5)	0.26632 (16)	0.0212 (3)	
C9	0.67714 (15)	0.74428 (6)	0.14834 (18)	0.0271 (3)	
H9	0.7073	0.7099	0.1173	0.033*	
C10	0.54048 (15)	0.75826 (6)	0.08056 (18)	0.0286 (3)	
H10	0.4782	0.7336	0.0035	0.034*	
C11	0.49232 (14)	0.81050 (6)	0.12697 (17)	0.0245 (3)	
H11	0.3987	0.8200	0.0787	0.029*	
C12	0.58172 (13)	0.84699 (5)	0.24163 (16)	0.0201 (3)	
H12	0.5483	0.8809	0.2711	0.024*	
C13	0.72615 (13)	0.83364 (5)	0.31691 (16)	0.0180 (3)	
C14	0.82019 (13)	0.86851 (5)	0.44328 (15)	0.0169 (3)	
C15	0.76793 (12)	0.92032 (5)	0.50670 (15)	0.0158 (3)	
C16	0.77237 (12)	0.97959 (5)	0.43933 (16)	0.0168 (3)	
H16A	0.7102	0.9837	0.3187	0.020*	
H16B	0.8659	0.9900	0.4446	0.020*	
C17	0.72303 (12)	1.01539 (5)	0.56842 (16)	0.0161 (3)	
H17	0.6460	1.0402	0.5030	0.019*	
C18	0.83723 (12)	1.04959 (5)	0.69927 (15)	0.0166 (3)	
C19	0.81301 (14)	1.10378 (5)	0.74894 (17)	0.0219 (3)	
H19	0.7262	1.1206	0.7005	0.026*	
C20	0.92081 (15)	1.13239 (6)	0.87233 (18)	0.0268 (3)	
H20	0.9077	1.1690	0.9069	0.032*	
C21	1.04791 (14)	1.10572 (6)	0.94313 (17)	0.0262 (3)	
H21	1.1217	1.1237	1.0271	0.031*	
C22	1.06228 (14)	1.05162 (6)	0.88576 (17)	0.0250 (3)	
H22	1.1479	1.0337	0.9337	0.030*	
C23	0.59907 (12)	0.97977 (5)	0.77386 (16)	0.0178 (3)	
C24	0.55832 (13)	0.92892 (6)	0.85831 (17)	0.0231 (3)	
H24A	0.5094	0.9410	0.9363	0.035*	
H24B	0.6403	0.9084	0.9245	0.035*	
H24C	0.4992	0.9048	0.7681	0.035*	

### Atomic displacement parameters (Å^2^)

**Table:**

	*U*^11^	*U*^22^	*U*^33^	*U*^12^	*U*^13^	*U*^23^
O1	0.0268 (5)	0.0234 (5)	0.0315 (5)	0.0046 (4)	0.0160 (4)	−0.0018 (4)
N1	0.0169 (5)	0.0145 (5)	0.0197 (5)	−0.0002 (4)	0.0066 (4)	−0.0027 (4)
N2	0.0174 (5)	0.0137 (5)	0.0208 (5)	−0.0006 (4)	0.0086 (4)	−0.0014 (4)
N3	0.0190 (5)	0.0223 (6)	0.0210 (5)	−0.0005 (4)	0.0037 (4)	−0.0005 (4)
C1	0.0212 (6)	0.0173 (6)	0.0190 (6)	0.0007 (5)	0.0108 (5)	0.0037 (5)
C2	0.0223 (6)	0.0196 (7)	0.0229 (6)	0.0005 (5)	0.0101 (5)	0.0018 (5)
C3	0.0217 (7)	0.0298 (8)	0.0242 (7)	−0.0001 (5)	0.0068 (5)	0.0035 (6)
C4	0.0209 (7)	0.0313 (8)	0.0320 (7)	0.0074 (6)	0.0107 (6)	0.0086 (6)
C5	0.0282 (7)	0.0224 (7)	0.0309 (7)	0.0087 (6)	0.0160 (6)	0.0061 (6)
C6	0.0251 (7)	0.0194 (7)	0.0229 (6)	0.0042 (5)	0.0134 (5)	0.0045 (5)
C7	0.0340 (8)	0.0168 (7)	0.0246 (7)	0.0045 (5)	0.0173 (6)	−0.0004 (5)
C8	0.0300 (7)	0.0186 (7)	0.0185 (6)	−0.0006 (5)	0.0130 (5)	−0.0001 (5)
C9	0.0391 (8)	0.0210 (7)	0.0247 (7)	−0.0014 (6)	0.0153 (6)	−0.0065 (5)
C10	0.0371 (8)	0.0271 (8)	0.0217 (7)	−0.0097 (6)	0.0097 (6)	−0.0082 (6)
C11	0.0256 (7)	0.0263 (7)	0.0207 (6)	−0.0040 (5)	0.0062 (5)	−0.0011 (5)
C12	0.0254 (7)	0.0174 (6)	0.0190 (6)	−0.0002 (5)	0.0095 (5)	0.0005 (5)
C13	0.0244 (6)	0.0157 (6)	0.0169 (6)	−0.0012 (5)	0.0106 (5)	0.0009 (5)
C14	0.0213 (6)	0.0156 (6)	0.0166 (6)	0.0002 (5)	0.0100 (5)	0.0019 (5)
C15	0.0130 (5)	0.0176 (6)	0.0158 (6)	−0.0014 (4)	0.0034 (4)	−0.0014 (5)
C16	0.0171 (6)	0.0168 (6)	0.0171 (6)	−0.0011 (5)	0.0062 (5)	−0.0004 (5)
C17	0.0171 (6)	0.0139 (6)	0.0177 (6)	0.0008 (5)	0.0064 (5)	0.0015 (5)
C18	0.0187 (6)	0.0174 (6)	0.0152 (6)	−0.0019 (5)	0.0078 (5)	0.0019 (5)
C19	0.0220 (6)	0.0212 (7)	0.0238 (6)	0.0001 (5)	0.0095 (5)	−0.0011 (5)
C20	0.0333 (8)	0.0222 (7)	0.0274 (7)	−0.0057 (6)	0.0132 (6)	−0.0081 (6)
C21	0.0259 (7)	0.0324 (8)	0.0200 (6)	−0.0104 (6)	0.0071 (5)	−0.0053 (6)
C22	0.0189 (7)	0.0320 (8)	0.0213 (6)	−0.0017 (5)	0.0026 (5)	0.0000 (6)
C23	0.0129 (6)	0.0228 (7)	0.0174 (6)	−0.0004 (5)	0.0045 (5)	−0.0026 (5)
C24	0.0215 (6)	0.0274 (7)	0.0237 (6)	−0.0015 (5)	0.0119 (5)	−0.0004 (5)

### Geometric parameters (Å, º)

**Table:**

O1—C23	1.2298 (15)	C10—H10	0.9300
N1—C15	1.2844 (15)	C11—C12	1.3651 (18)
N1—N2	1.3957 (13)	C11—H11	0.9300
N2—C23	1.3578 (15)	C12—C13	1.4290 (18)
N2—C17	1.4862 (15)	C12—H12	0.9300
N3—C22	1.3406 (17)	C13—C14	1.4075 (17)
N3—C18	1.3420 (16)	C14—C15	1.4874 (16)
C1—C14	1.4071 (17)	C15—C16	1.5052 (17)
C1—C2	1.4321 (18)	C16—C17	1.5402 (16)
C1—C6	1.4355 (18)	C16—H16A	0.9700
C2—C3	1.3630 (18)	C16—H16B	0.9700
C2—H2	0.9300	C17—C18	1.5168 (17)
C3—C4	1.4244 (19)	C17—H17	0.9800
C3—H3	0.9300	C18—C19	1.3852 (17)
C4—C5	1.359 (2)	C19—C20	1.3871 (19)
C4—H4	0.9300	C19—H19	0.9300
C5—C6	1.4305 (18)	C20—C21	1.382 (2)
C5—H5	0.9300	C20—H20	0.9300
C6—C7	1.3966 (19)	C21—C22	1.3806 (19)
C7—C8	1.3923 (19)	C21—H21	0.9300
C7—H7	0.9300	C22—H22	0.9300
C8—C9	1.4274 (19)	C23—C24	1.5009 (17)
C8—C13	1.4396 (17)	C24—H24A	0.9600
C9—C10	1.358 (2)	C24—H24B	0.9600
C9—H9	0.9300	C24—H24C	0.9600
C10—C11	1.420 (2)		
			
C15—N1—N2	107.53 (10)	C12—C13—C8	118.40 (11)
C23—N2—N1	122.08 (10)	C1—C14—C13	121.05 (11)
C23—N2—C17	124.83 (10)	C1—C14—C15	119.46 (11)
N1—N2—C17	113.08 (9)	C13—C14—C15	119.39 (11)
C22—N3—C18	117.04 (11)	N1—C15—C14	119.44 (11)
C14—C1—C2	122.05 (11)	N1—C15—C16	114.67 (10)
C14—C1—C6	119.33 (11)	C14—C15—C16	125.87 (10)
C2—C1—C6	118.58 (11)	C15—C16—C17	102.42 (9)
C3—C2—C1	120.69 (12)	C15—C16—H16A	111.3
C3—C2—H2	119.7	C17—C16—H16A	111.3
C1—C2—H2	119.7	C15—C16—H16B	111.3
C2—C3—C4	120.79 (13)	C17—C16—H16B	111.3
C2—C3—H3	119.6	H16A—C16—H16B	109.2
C4—C3—H3	119.6	N2—C17—C18	110.17 (9)
C5—C4—C3	120.07 (12)	N2—C17—C16	100.90 (9)
C5—C4—H4	120.0	C18—C17—C16	114.22 (10)
C3—C4—H4	120.0	N2—C17—H17	110.4
C4—C5—C6	121.29 (12)	C18—C17—H17	110.4
C4—C5—H5	119.4	C16—C17—H17	110.4
C6—C5—H5	119.4	N3—C18—C19	123.05 (11)
C7—C6—C5	122.26 (12)	N3—C18—C17	115.51 (10)
C7—C6—C1	119.24 (12)	C19—C18—C17	121.42 (11)
C5—C6—C1	118.48 (12)	C18—C19—C20	118.69 (12)
C8—C7—C6	121.84 (12)	C18—C19—H19	120.7
C8—C7—H7	119.1	C20—C19—H19	120.7
C6—C7—H7	119.1	C21—C20—C19	119.04 (13)
C7—C8—C9	122.02 (12)	C21—C20—H20	120.5
C7—C8—C13	119.38 (12)	C19—C20—H20	120.5
C9—C8—C13	118.57 (12)	C22—C21—C20	118.17 (12)
C10—C9—C8	121.28 (12)	C22—C21—H21	120.9
C10—C9—H9	119.4	C20—C21—H21	120.9
C8—C9—H9	119.4	N3—C22—C21	124.00 (12)
C9—C10—C11	120.18 (12)	N3—C22—H22	118.0
C9—C10—H10	119.9	C21—C22—H22	118.0
C11—C10—H10	119.9	O1—C23—N2	119.56 (11)
C12—C11—C10	120.81 (13)	O1—C23—C24	123.16 (11)
C12—C11—H11	119.6	N2—C23—C24	117.27 (11)
C10—C11—H11	119.6	C23—C24—H24A	109.5
C11—C12—C13	120.73 (12)	C23—C24—H24B	109.5
C11—C12—H12	119.6	H24A—C24—H24B	109.5
C13—C12—H12	119.6	C23—C24—H24C	109.5
C14—C13—C12	122.48 (11)	H24A—C24—H24C	109.5
C14—C13—C8	119.07 (11)	H24B—C24—H24C	109.5
			
C15—N1—N2—C23	174.55 (11)	C8—C13—C14—C1	1.11 (17)
C15—N1—N2—C17	−6.35 (13)	C12—C13—C14—C15	2.16 (17)
C14—C1—C2—C3	178.56 (12)	C8—C13—C14—C15	−175.24 (10)
C6—C1—C2—C3	0.70 (18)	N2—N1—C15—C14	179.49 (10)
C1—C2—C3—C4	1.56 (19)	N2—N1—C15—C16	−1.87 (13)
C2—C3—C4—C5	−1.6 (2)	C1—C14—C15—N1	−95.42 (14)
C3—C4—C5—C6	−0.7 (2)	C13—C14—C15—N1	80.99 (14)
C4—C5—C6—C7	−175.71 (12)	C1—C14—C15—C16	86.11 (15)
C4—C5—C6—C1	2.95 (19)	C13—C14—C15—C16	−97.48 (14)
C14—C1—C6—C7	−2.10 (18)	N1—C15—C16—C17	8.65 (13)
C2—C1—C6—C7	175.81 (11)	C14—C15—C16—C17	−172.82 (11)
C14—C1—C6—C5	179.20 (11)	C23—N2—C17—C18	69.14 (14)
C2—C1—C6—C5	−2.89 (17)	N1—N2—C17—C18	−109.93 (11)
C5—C6—C7—C8	178.87 (12)	C23—N2—C17—C16	−169.81 (11)
C1—C6—C7—C8	0.22 (19)	N1—N2—C17—C16	11.12 (12)
C6—C7—C8—C9	−175.89 (12)	C15—C16—C17—N2	−10.80 (11)
C6—C7—C8—C13	2.32 (19)	C15—C16—C17—C18	107.34 (11)
C7—C8—C9—C10	−179.99 (12)	C22—N3—C18—C19	0.75 (18)
C13—C8—C9—C10	1.78 (19)	C22—N3—C18—C17	−177.77 (10)
C8—C9—C10—C11	−0.3 (2)	N2—C17—C18—N3	73.20 (13)
C9—C10—C11—C12	−0.9 (2)	C16—C17—C18—N3	−39.52 (14)
C10—C11—C12—C13	0.39 (19)	N2—C17—C18—C19	−105.35 (12)
C11—C12—C13—C14	−176.25 (11)	C16—C17—C18—C19	141.94 (11)
C11—C12—C13—C8	1.16 (18)	N3—C18—C19—C20	0.06 (19)
C7—C8—C13—C14	−2.97 (17)	C17—C18—C19—C20	178.49 (11)
C9—C8—C13—C14	175.30 (11)	C18—C19—C20—C21	−0.82 (19)
C7—C8—C13—C12	179.53 (11)	C19—C20—C21—C22	0.74 (19)
C9—C8—C13—C12	−2.20 (17)	C18—N3—C22—C21	−0.84 (19)
C2—C1—C14—C13	−176.43 (11)	C20—C21—C22—N3	0.1 (2)
C6—C1—C14—C13	1.41 (18)	N1—N2—C23—O1	−179.52 (10)
C2—C1—C14—C15	−0.08 (18)	C17—N2—C23—O1	1.48 (18)
C6—C1—C14—C15	177.76 (10)	N1—N2—C23—C24	−0.39 (16)
C12—C13—C14—C1	178.50 (11)	C17—N2—C23—C24	−179.38 (10)

### Hydrogen-bond geometry (Å, º)

**Table:**

*D*—H···*A*	*D*—H	H···*A*	*D*···*A*	*D*—H···*A*
C12—H12···O1^i^	0.93	2.42	3.2745 (16)	153
C24—H24*A*···O1^ii^	0.96	2.58	3.5265 (16)	167

Symmetry codes: (i) −*x*+1, −*y*+2, −*z*+1; (ii) −*x*+1, −*y*+2, −*z*+2.
